# Mediating role of preterm birth in the relationship between maternal disease and infant development

**DOI:** 10.1186/s12884-025-08268-7

**Published:** 2025-11-07

**Authors:** Shan Tan, Yiduo Wang, Changshi Tang, Shizhou Li, Jiang Mei

**Affiliations:** 1 Department of Pediatrics, The Third Xiangya Hospital, Central South University, Changsha, China; 2 The Nick Davey Laboratory, Division of Surgery, Department of Surgery and Cancer, Faculty of Medicine, Sir Michael Uren Hub, Imperial College London, White City Campus, 86 Wood Lane, London, W12 0BZ UK; 3 Chancellor’s Building, University of Edinburgh, Edinburgh, EH16 4SB UK; 4 The VOC Laboratory, Division of Surgery, Department of Surgery and Cancer, Faculty of Medicine, Imperial College London, Commonwealth Building, Hammersmith Campus, London, W12 0NN UK; 5 Nursing Department, The Third Xiangya Hospital, Central South University, Changsha, Hunan China

**Keywords:** Mediation analysis, Preterm birth, Body mass index, Pregnancy-Induced hypertension, Gestational diabetes mellitus, Neurodevelopmental impairment

## Abstract

**Background:**

Preterm birth is a major adverse perinatal outcome and may act as a mediator linking maternal disease to impaired infant growth and neurodevelopment. However, the mediating role of preterm birth has not been well explored in relation to maternal diseases. This study aimed to investigate whether preterm birth mediates the association between maternal diseases and infant outcomes including Body Mass Index, the Neonatal Behavioral Neurological Assessment, and the Gesell Development Schedule.

**Methods:**

This study recruited a total of 2000 mother-child pairs from the Pediatric Healthcare Centre at the Third Xiangya Hospital. Maternal diseases, including gestational diabetes mellitus, pregnancy-induced hypertension, anaemia, hypothyroidism, hyperthyroidism, and thrombocytopenia, were assessed through hospital records and parental questionnaires verified against medical records. Infant outcomes were evaluated by trained pediatric nurses blinded to maternal conditions, using anthropometric measurements (Body Mass Index) and standardized neurodevelopmental tools (Neonatal Behavioral Neurological Assessment and Gesell Development Schedule). Mediation analysis with 1,000-sample bootstrapping was applied to quantify the indirect effects of preterm birth.

**Results:**

Preterm birth significantly mediated the association between Pregnancy-Induced Hypertension and Neonatal Behavioral Neurological Assessment (Indirect effect = -0.653, 95% CI: -0.98 – -0.36, *p* < 0.001). Preterm birth also significantly mediated the relationship between Gestational Diabetes Mellitus and Body Mass Index (Indirect effect = − 0.046, 95% CI: -0.08 – -0.01, *p* = 0.01).

**Conclusions:**

Preterm birth may act as a mediator between maternal diseases and infant growth and neurodevelopmental outcomes. The results support the feasibility and value of using mediation analysis in maternal–infant research. Findings highlight the importance of early prenatal screening, prevention, and management of preterm birth in pregnancies complicated by maternal disease. Future research is warranted to employ longitudinal neurodevelopmental monitoring in affected infants.

**Supplementary Information:**

The online version contains supplementary material available at 10.1186/s12884-025-08268-7.

## Background

Maternal health conditions significantly impact both maternal well-being and infant growth and development, contributing to adverse pregnancy outcomes [1]. These conditions include a range of short and long-term health issues that arise during pregnancy, childbirth, or the postnatal period [2]. Common maternal diseases include anaemia (37%), gestational thrombocytopenia (12%), gestational diabetes mellitus (GDM; 8.3%), hypothyroidism (7.5%), pregnancy-induced hypertension (PIH; 7.3%), and hyperthyroidism (0.4%) [3–8]. These conditions have been linked to poor foetal and neonatal outcomes; for example, GDM is associated with an increased risk of foetal macrosomia, while anaemia and PIH are linked to low birth weight [9, 10]. These figures emphasize the critical role of maternal health in determining the growth and development outcomes of infants over the short and long term.

Preterm birth which is defined as delivery before 37 completed weeks of gestation [11], is a major independent risk factor for impaired infant growth and development [12, 13]. Globally, approximately 10.6% of live births are preterm, making preterm birth one of the leading causes of death in children under five years of age [14]. Additionally, preterm birth is associated with a higher risk of complications such as developmental delays, intellectual disabilities, and chronic health problems. For example, studies in France have reported significant neurodevelopmental impairment in up to 38.5% of preterm infants [15, 16]. The evidence suggests that preterm birth not only contributes to perinatal mortality but may also be an important determinant of early-life health trajectories and socioeconomic burden.

Studies have shown that preterm birth associates with various maternal diseases, including PIH, GDM, systemic lupus erythematosus, hyperthyroidism, cardiovascular disease, asthma, and renal disorders [17, 18]. It also appears that the co-existence of maternal disease and preterm birth has been associated with more severe adverse outcomes in infants compared to either risk factor alone. For example, infants exposed to both preterm birth and maternal disease are at an increased risk for growth delays, neurodevelopmental impairments, and metabolic disorders [19]. Maternal risk factors can induce systemic inflammation, placental dysfunction, or uterine overactivity, which trigger preterm birth [20–22]. Preterm birth can impair organ maturation and growth trajectories of infants, which result in adverse outcomes [23]. This suggests that preterm birth may function as a contributor mediating detrimental effects of maternal diseases on infants’ outcomes.

Mediation analysis has been widely applied in the domain of maternal and child health. The mediating effect of preterm birth has been investigated on the association between GDM and psychiatric disorders including the incidence of autism, attention-deficit/hyperactivity disorder, and intellectual disability [24]. There are also studies targeting on the mediating effects of preterm birth between preeclampsia, cerebral palsy, and poor school performance [25, 26]. However, studies have largely focused on pre-eclampsia and examined outcomes after adjusting for preterm birth rather than explicitly modelling mediation effect. Meanwhile, these studies treated preterm as a composition in a set of complications instead of an independent variable, and the outcome variables lack physical growth reference. The detailed interpretation of the mediating effects of preterm birth between comprehensive maternal diseases and both neurological and physical development of infants is needed.

This study aims to clarify how preterm birth mediates the impact of maternal disease on infant growth and development. Identifying this mediating role may inform clinical management, preventive strategies, and public health policy to improve maternal - infant health outcomes.

## Methods

### Study design

This retrospective cohort study included 3,303 mother–infant pairs recruited at the Paediatric Healthcare Centre, Third Xiangya Hospital (2021–2023). Maternal exposures were defined by the incidence of specific prenatal diseases documented in the hospital database. GDM, hypothyroidism, anaemia, PIH, thrombocytopenia, and hyperthyroidism were included with incidence above 1% in the recruitment cohort. Preterm birth (< 37 weeks) was selected as the mediator due to its high global incidence of 10.6% [14] and its established adverse effects on infant outcomes. Infant outcome measures were identified complying with the practical standard for infant development evaluation in China [27–29]. All the assessment examinations were performed by the paediatric nurses blinded to maternal conditions. The study adhered to the ethical principles of the Declaration of Helsinki (2013) and complied with STROBE (Strengthening the Reporting of Observational Studies in Epidemiology) guidelines for observational research. Parental or legal guardian consent was obtained for all infant participants.

### Procedures

**Fig. 1:**
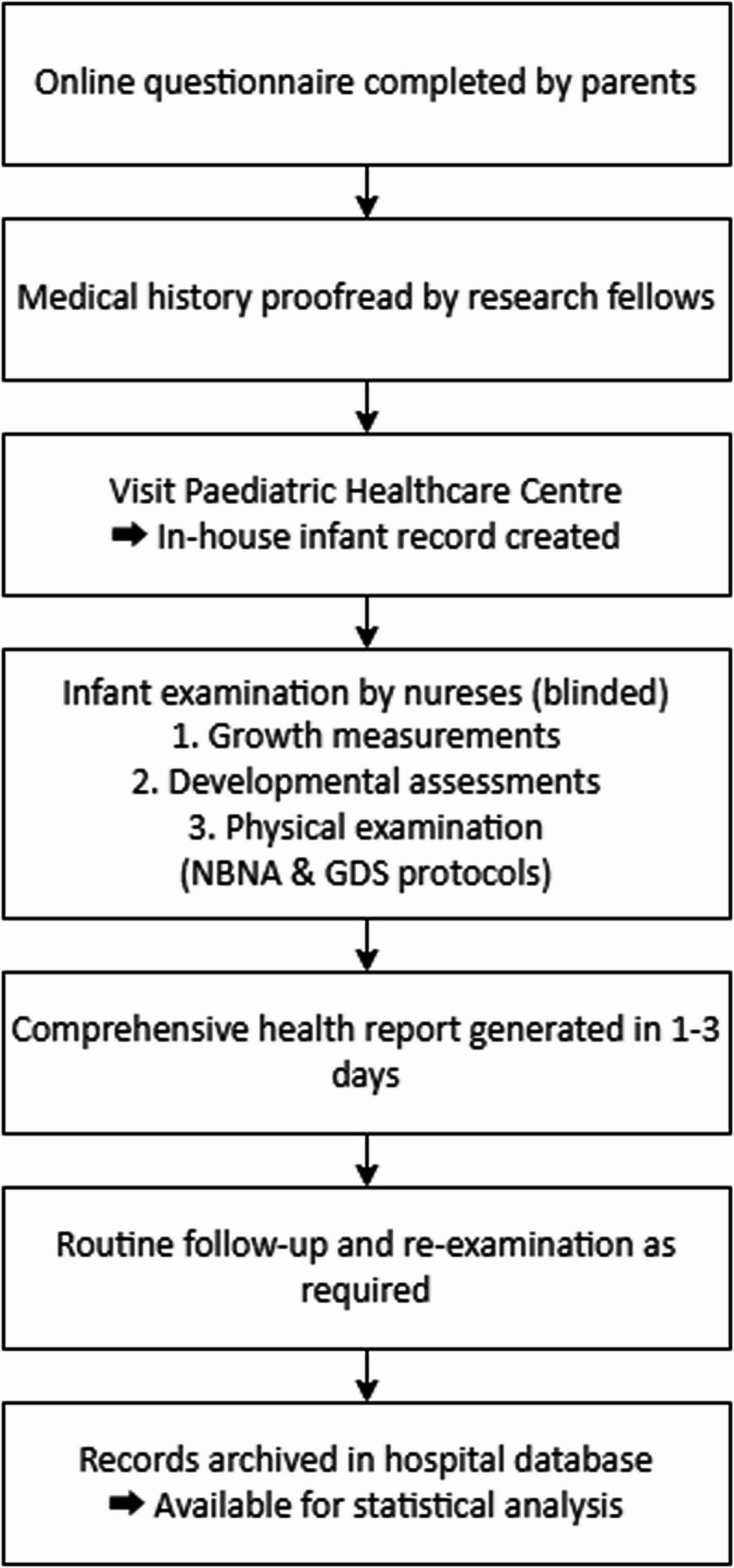
Clinical assessment workflow for research

At recruitment, parents completed an online questionnaire (Additional file 1) prior to the child’s health check. Information on maternal medical history provided by the parents from the questionnaires was cross-checked by the clinical research fellows referred to previous records from matching hospitals. Each family was then registered at the Paediatric Healthcare Centre, where an in-house record was created for the infant. Assessment examination of infants was then conducted by the nurses blinded of the participants’ conditions, including growth measurements, developmental assessments, physical examinations. The examination procedure followed the Neonatal Behavioral Neurological Assessment (NBNA; Additional file 2) and Gesell Developmental Schedule (GDS; Additional file 3). Within one to three working days, a comprehensive health report was issued, followed by routine health follow-up and re-examination as required. All participant records were automatically archived in the hospital database for subsequent statistical analysis. The clinical assessment workflow is illustrated in Fig. 1.

### Participants

**Fig. 2:**
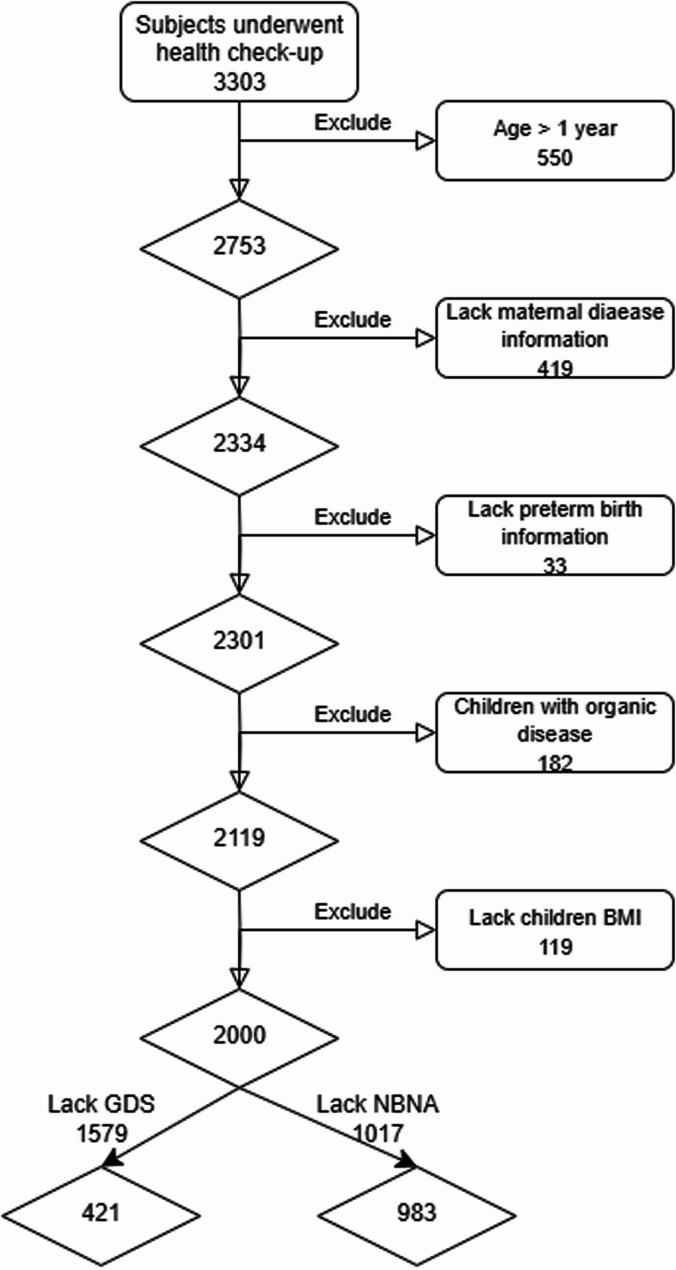
Participant recruitment workflow

A total of 3303 mother-infant pairs were randomly recruited at their first health check-up at the Pediatric Healthcare Centre. Inclusion criteria were: (i) infants aged 365 days at examination; and (ii) mothers who completed a survey detailing the infant’s birth history and maternal prenatal conditions. Exclusion criteria were: (i) incomplete data on infant birth or maternal prenatal disease; (ii) infants with a history of surgery or diagnosed organic diseases (e.g., neurological, cardiovascular, respiratory, gastrointestinal, metabolic, musculoskeletal, endocrine, genetic or chromosomal, renal or urological, haematological, or immune); and (iii) infants receiving non-routine medications. Infants with organic diseases were excluded because such conditions could independently influence growth and neurodevelopment. It can interfere the mediating role of preterm birth. The recruitment and classification process is shown in Fig. 2.

### Data Collection

Clinical data was obtained from structured questionnaires and physical assessment. Questionnaires recorded: (i) infant data including age, sex, gestational age, birth weight, birth length/height, and preterm birth status; (ii) maternal data including age, body type, and prenatal conditions such as PIH, GDM, anaemia, hypothyroidism, hyperthyroidism, and thrombocytopenia. Age was defined as chronological age [30, 31]. The medical history was crossed-checked by clinical research fellows referred to the previous diagnostic record from matching hospitals. Physical assessments including infant length/height, weight, the NBNA score, and GDS score were performed by trained paediatric nurses who were blinded to maternal disease status.

### Exposure variable

Maternal diseases were clinically diagnosed during routine prenatal check-ups. GDM was defined by a 75 g oral glucose tolerance test at 24–28 weeks’ gestation [32]. PIH was defined as systolic blood pressure ≥ 140 mmHg and/or diastolic blood pressure ≥ 90 mmHg measured on at least two occasions after 20 weeks’ gestation [33]. Thyroid disorders including hyperthyroidism and hypothyroidism were defined based on serum thyroid-stimulating hormone and free thyroxine levels measured during pregnancy [34]. Anaemia was defined as haemoglobin thresholds < 110 g/L in the first trimesters or < 105 g/L in the second and third trimester [35]. Maternal diseases were recorded for the index pregnancy irrespective of any previous occurrence.

### Mediation variable

Preterm birth status was determined from gestational age documented in the infant’s medical records.

### Outcome variables

Postnatal BMI was calculated as weight in kilograms divided by the square of height in meters (kg/m^2^).

The NBNA is a standardised tool widely used in China to evaluate the behavioural and neurological status of newborns [27]. It consists of five domains: behavioural ability (6 items), passive muscle tone (4 items), active muscle tone (4 items), primitive reflexes (3 items), and a general assessment (3 items). Each item is scored from 0 to 2, with a maximum total score of 40. A score below 35 indicates abnormal neurological function. Assessments were conducted between feedings, in a quiet, dimly lit room with a temperature maintained between 22 °C and 27 °C.

The GDS is used to assess developmental level and screen for intellectual disabilities in children aged 0–6 years [36]. It evaluates five domains: adaptive behaviour, gross motor skills, fine motor skills, language, and personal-social behaviour. Developmental delay is categorised as mild (55–75), moderate (25–39), and extremely severe (< 25) based on standardised scores.

### Covariates

Covariates included infants’ age, sex, and birth weight, as well as maternal pre-pregnant body type categorised as underweight (BMI < 18.5), normal (BMI 18.5–23.9), and overweight (BMI > 24.0) [37].

### Statistical analysis

All statistical analyses were performed using R software (version 3.6.3). Continuous variables were expressed as mean (standard deviation) or median [interquartile range] and compared between two groups using either the t-test or Mann–Whitney U test as appropriate. Categorical variables were described as counts (percentages) and compared between groups using the chi-square or Fisher’s exact test. The analytical process involved data pre-processing, univariate and multivariate analyses, and mediation analyses. Missing data were handled by listwise deletion. Participants with complete data were included for each outcome containing GDS (*n* = 421) and NBNA (*n* = 983). Univariate and multivariate analyses were performed with the *Stats* package; mediation analyses used the *Mediation* package. Mediation analysis was conducted using a bootstrapping method with 1,000 resamples. A 95% bias-corrected confidence interval was calculated to assess whether the mediator significantly contributed to the relationship between the independent and dependent variables.

## Results

### Sample descriptives

**Fig. 3:**
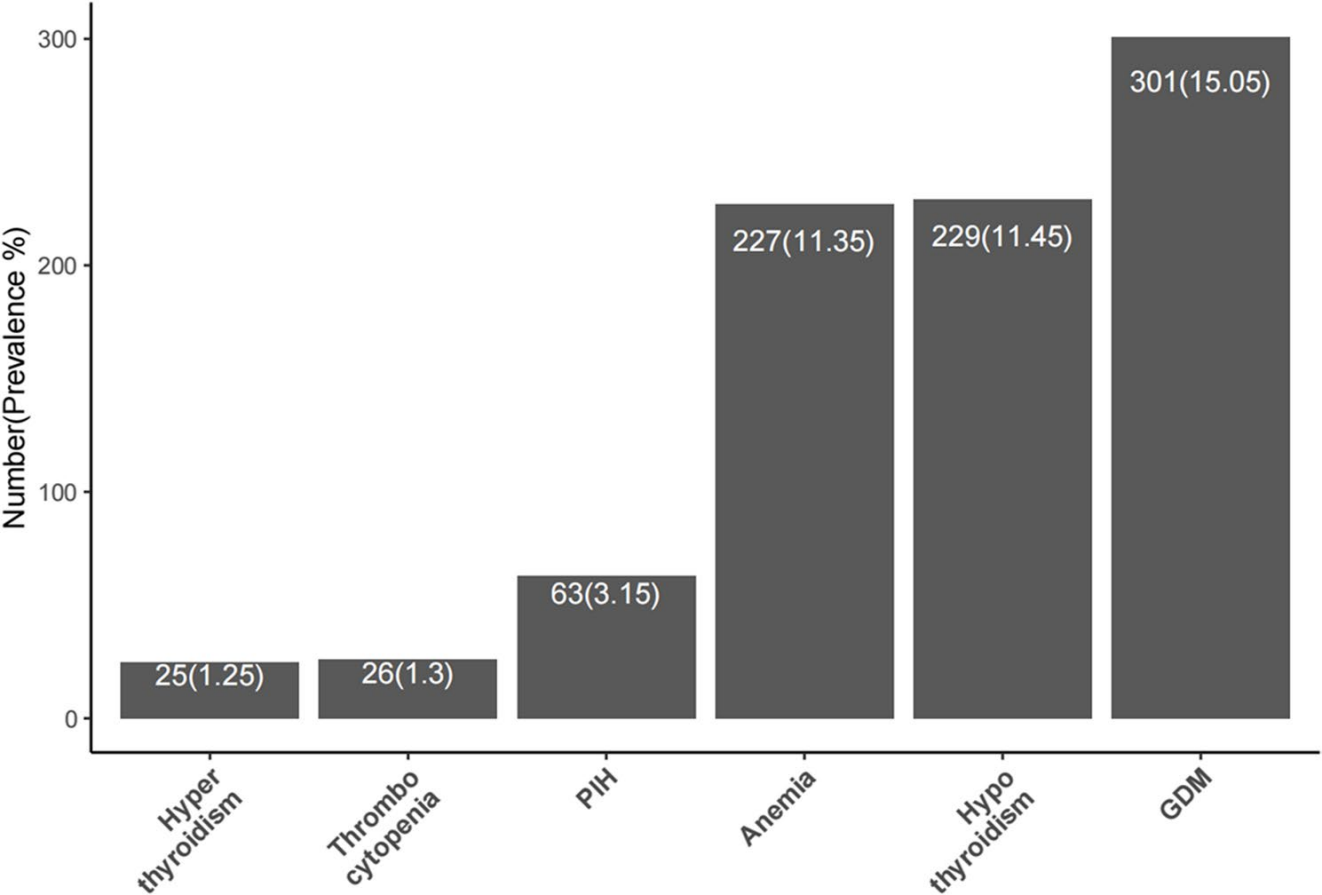
Incidence of maternal diseases among recruited subjects

A total of 2000 mother-infant pairs were included in the analysis. Among them, 1601 (80.05%) mothers had a normal pre-pregnant body type, with the BMI of 18.5–23.9 kg/m^2^. The incidence of preterm birth was 140 cases (7.0%). The incidence of each maternal disease among the study population is shown in Fig. 3. Infant growth was assessed by BMI, with a mean of 16.15 ± 1.94 kg/m². Neurodevelopment was evaluated using the NBNA and the GDS, yielding mean scores of 38.80 ± 1.81 and 90.52 ± 10.48, respectively. Other covariates including infant sex, birth weight (g), and infant chronological age (days) are summarised in Table 1.

**Table 1:** Descriptive characteristics of study variables (Exposures, Mediator, Outcomes, and Covariates)

Exposure		Frequency	%
GDM	Yes	301	15.05
	No	1699	84.95
PIH	Yes	63	3.15
	No	1937	96.85
Hyperthyroidism	Yes	25	1.25
	No	1975	98.75
Thrombocytopenia	Yes	26	1.30
	No	1974	98.70
Hypothyroidism	Yes	229	11.45
	No	1771	88.55
Anaemia	Yes	227	11.35
	No	1773	88.65
Mediator		Frequency	%
Preterm birth	Yes	140	7.0
	No	1860	93.0
Outcome		Mean	SD
BMI (Kg/m^2^)		16.15	1.94
NBNA		38.80	1.81
GDS		90.52	10.48
Covariate		Frequency	%
Pre-pregnant body type	Underweight	130	6.50
	Normal	1601	80.05
	Obese	269	13.45
Infant sex	Male	1054	52.70
	Female	946	47.30
Birth weight (g)	≤ 2500	171	8.55
	2500–4000	1785	89.25
	> 4000	44	2.20
Infant age (days)	≤ 90	1081	54.05
	91–180	404	20.20
	181–365	515	25.75

Abbreviation *GDM* Gestational Diabetes Mellitus, *PIH *Pregnancy-Induced Hypertension, *BMI* Body Mass Index, *NBNA* Neonatal Behavioural Neurological Assessment, *GDS* Gesell Development Schedule

### Univariate analysis

GDM was significantly associated with preterm birth (*P* < 0.001) and infant neurodevelopmental outcome measured by the NBNA score (*P* = 0.055). PIH was significantly associated with preterm birth (*P* < 0.001) and infant neurodevelopmental outcome measured by GDS score (*P* = 0.013) and NBNA score (*P* = 0.099). Preterm birth was significantly associated with both NBNA score and GDS score (all *P* < 0.001). Maternal thrombocytopenia, hypothyroidism, and anaemia during pregnancy showed no significant associations with preterm birth, infant BMI, NBNA score, or GDS score. Although maternal hyperthyroidism was significantly associated with infant BMI (*P* = 0.05), the small number of affected mothers (*n* = 25) limited further analysis. Therefore, only GDM and PIH were retained as exposure variables in the multivariate analyses. Detailed univariate results are presented in Table 2.

**Table 2:** Univariate analysis of the relationships between maternal disease, PTB, and infant growth and developmental outcomes

Exposure	Outcome	Exposure=no	Exposure=yes	p
GDM	preterm birth, n (%)			<0 .001
	no	1596 (93.9)	264 (87.7)	
	yes	103 (6.1)	37 (12.3)	
	BMI, Median (IQR)	16.1(14.8, 17.4)	16.0 (14.7, 17.4)	0.246
	NBNA, Median (IQR)	40 (38, 40)	40 (37, 40)	0.055
	GDS, Median (IQR)	91.7 (86.3, 97.1)	90.9 (83.3, 97.1)	0.284
PIH	preterm birth, n (%)			< 0.001
	no	1814 (93.6)	46 (73.0)	
	yes	123 (6.4)	17 (27.0)	
	BMI, Median (IQR)	16.0 (14.8, 17.4)	16.1 (15.1, 17.4)	0.518
	NBNA, Median (IQR)	40 (38, 40)	39 (35, 40)	0.099
	GDS, Median (IQR)	91.8 (85.6, 97.7)	86.5 (83.2, 91.4)	0.013
Hyperthyroidism	preterm birth, n (%)			1.0^a^
	no	1837 (93.0)	23 (92.0)	
	yes	138 (7.0)	2 (8.0)	
	BMI, Median (IQR)	16.1 (14.8, 17.4)	15.3(14.5, 16.0)	0.050
	NBNA, Median (IQR)	40 (38, 40)	39 (37, 40)	0.194
	GDS, Median (IQR)	91.6 (85.2, 97.1)	95.5 (90.1, 96.6)	0.717
Thrombocytopenia	preterm birth, n (%)			1.0^a^
	no	1836 (93.0)	24 (92.3)	
	yes	138 (7.0)	2 (7.7)	
	BMI, Median (IQR)	16.1(14.8, 17.4)	15.5 (15.0, 17.6)	0.532
	NBNA, Median (IQR)	40 (38, 40)	40 (38, 40)	0.459
	GDS, Median (IQR)	91.6 (85.2, 97.1)	94.0 (87.2, 94.7)	0.964
Hypothyroidism	preterm birth, n (%)			0.897
	no	1648 (93)	212 (93)	
	yes	123 (7)	17 (7)	
	BMI, Median (IQR)	16.1 (14.8, 17.4)	15.9 (14.8, 17.3)	0.394
	NBNA, Median (IQR)	40 (38, 40)	40 (38, 40)	0.569
	GDS, Median (IQR)	91.8(85.2, 97.8)	90.9 (85.6, 95.4)	0.397
Anaemia	preterm birth, n (%)			0.282
	no	1645 (92.8)	215 (94.7)	
	yes	128 (7.2)	12 (5.3)	
	BMI, Median (IQR)	16.0 (14.8, 17.4)	16.4(14.9, 17.6)	0.158
	NBNA, Median (IQR)	40 (38, 40)	40 (38, 40)	0.732
	GDS, Median (IQR)	91.2 (85.0, 96.9)	93.5 (87.6, 99.4)	0.220

Abbreviations* PTB* Preterm Birth, *GDM* Gestational Diabetes Mellitus, *PIH* Pregnancy-Induced Hypertension, *BMI *Body Mass Index, *NBNA *Neonatal Behavioural Neurological Assessment, GDS: Gesell Development Schedule

### Multivariate regression analysis

To explore the mediating role of preterm birth, multivariate regression analyses were performed, adjusting for covariates including infant age, sex, birth weight, pre-pregnant body type, and maternal hyperthyroidism. In these models, GDM (OR = 1.967; *P* = 0.001) and PIH (OR = 4.758; *P* < 0.001) were both significantly associated with an increased risk of preterm birth. Preterm birth was significantly associated with lower infant BMI (β = − 0.553; *P* = 0.004), GDS score (β = − 17.133; *P* < 0.001), and NBNA score (β = − 3.817; *P* < 0.001). GDM was associated with lower infant BMI (β = − 0.208; *P* = 0.047), but its effect on BMI among preterm infants was not significant (β = − 0.184, *P* = 0.079). PIH was significantly associated with NBNA score (β = − 0.259, *P* = 0.012), although this effect was not significant among preterm infants (*P* > 0.05). The associations of GDM and PIH with infant outcomes (BMI, GDS score, and NBNA score) were weaker after adjusting for preterm birth. Since GDM and PIH were both significantly associated with preterm birth, and preterm birth with all three outcomes, we hypothesised that preterm birth may function as a mediator of these associations. Full multivariate regression results are provided in Table 3.

**Table 3:** Multivariate analysis of GDM, PIH, and PTB on BMI, GDS, and NBNA after adjusting for covariates

Models	GDM		PIH		PTB	
	β (95% CI)	P value	β (95% CI)	P value	β (95%CI)	P value
BMI model	−0.208(−0.413~−0.003)	0.047	−0.035(−0.456 ~ 0.387)	0.871		
BMI model + PTB	−0.184(−0.390 ~ 0.022)	0.079	−0.035(−0.456 ~ 0.386)	0.869	−0.553(−0.927~−0.180)	0.004
GDS score model	−1.643(−4.234 ~ 0.949)	0.213	−4.081(−8.708 ~ 0.546)	0.084		
GDS score model + PTB	−0.614(−2.921 ~ 1.693)	0.601	−1.781(−5.908 ~ 2.346)	0.397	−17.133(−20.295~−13.971)	< 0 0.001
NBNA score model	−0.259(−0.576 ~ 0.057)	0.108	−1.003(−1.784~−0.222)	0.012		
NBNA score model + PTB	−0.216(−0.503 ~ 0.071)	0.141	−0.326(−1.040 ~ 0.388)	0.370	−3.817(−4.330~−3.303)	< 0.001
	OR (95% CI)	P value	OR (95% CI)	P value		
PTB model	1.967(1.286 ~ 2.947)	0.001	4.758(2.508 ~ 8.669)	< 0.001		

The basic BMI model was adjusted for age, sex, birth weight, maternal body type, and hyperthyroidism. The basic GDS score model, and basic NBNA score model were adjusted for age and sex. All other preterm birth models were based on the basic model, with the addition of a preterm birth indicator

Abbreviations* PTB* Preterm Birth, *GDM* Gestational Diabetes Mellitus, *PIH* Pregnancy-Induced Hypertension, *BMI* Body Mass Index,* NBNA* Neonatal Behavioural Neurological Assessment, *GDS* Gesell Development Schedule

### Mediation analysis

**Fig. 4:**
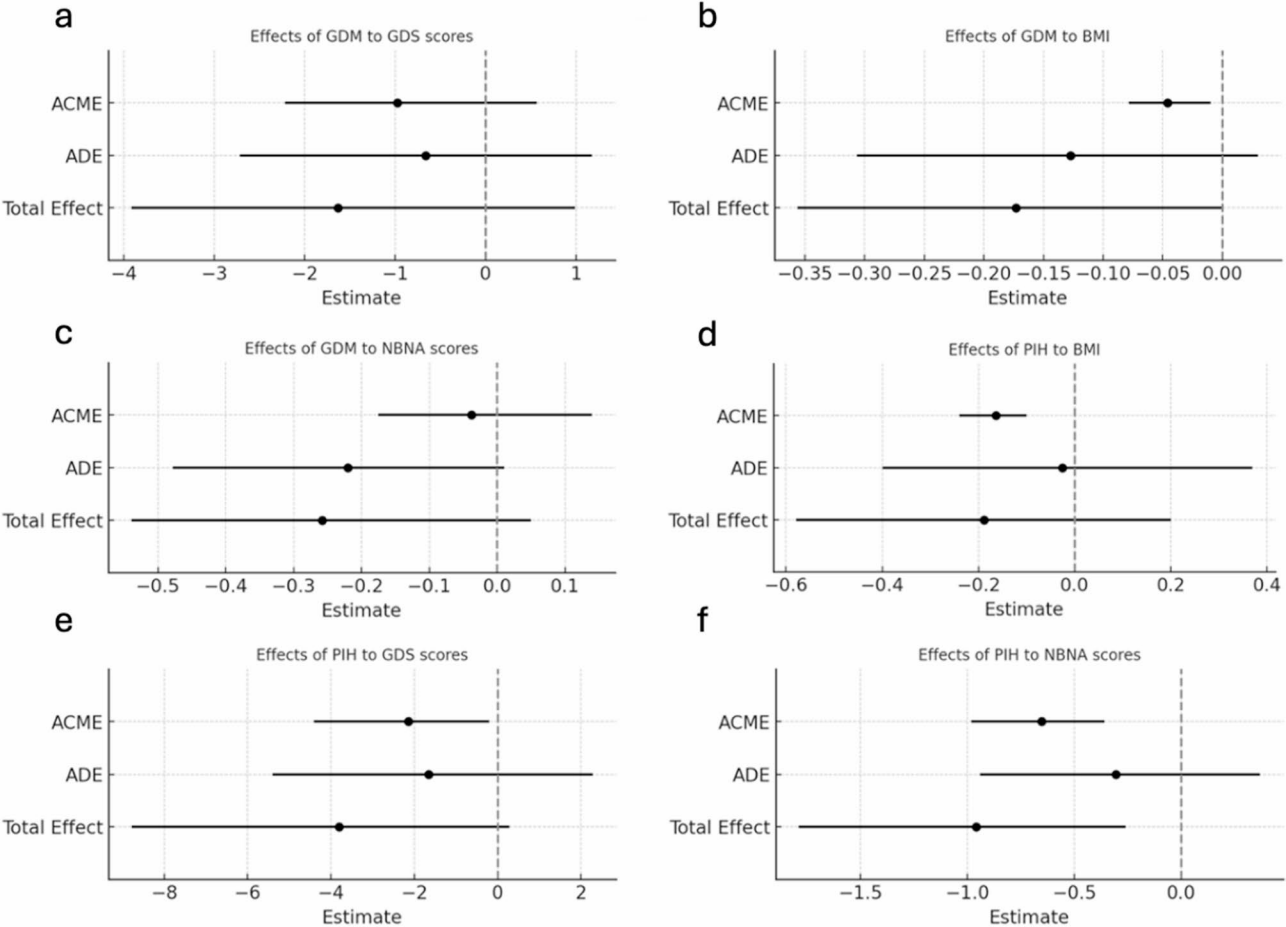
Mediating effects of GDM and PIH on infant outcomes through preterm birth

Mediation analyses further indicated indirect effects of preterm birth on infant outcomes via GDM and PIH (Table 4; Fig. 4). After adjustment for infant age, sex, and PIH, the total effect of GDM on infant BMI was significant (Total Effect = − 0.1733; *P* = 0.05), while the direct effect was not significant (Average Direct Effect [ADE] = − 0.1275; *P* > 0.05). The indirect effect via preterm birth was significant (Average Causal Mediation Effect [ACME] = − 0.0459; *P* = 0.01), indicating that preterm birth completely mediated the effect of GDM on BMI. Similarly, after adjustment for infant age, sex, and GDM, the total effect of PIH on NBNA score was significant (Total Effect = − 0.959; *P* < 0.001), whereas direct effect was not significant (ADE = − 0.305; *P* > 0.05). The indirect effect via preterm birth was significant (ACME = − 0.653, *P* < 0.001), demonstrating that preterm birth completely mediated the effect of PIH on infant NBNA score.

**Table 4:** Mediation analysis of preterm birth on infant growth and development

Models	Effect	Estimate	95% CI Lower	95% CI Upper	*p*-value
GDM to BMI	ACME	−0.046	−0.08	−0.01	0.01 **
	ADE	−0.128	−0.31	0.03	0.2
	Total Effect	−0.173	−0.36	0	0.05 *
PIH to BMI	ACME	−0.163	−0.24	−0.10	< 0.001 ***
	ADE	−0.025	−0.40	0.37	0.91
	Total Effect	−0.189	−0.58	0.20	0.39
GDM to GDS score	ACME	−0.975	−2.21	0.57	0.18
	ADE	−0.658	−2.72	1.18	0.59
	Total Effect	−1.633	−3.92	0.99	0.21
PIH to GDS score	ACME	−2.148	−4.40	−0.21	0.04 *
	ADE	−1.661	−5.40	2.29	0.49
	Total Effect	−3.809	−8.78	0.28	0.11
GDM to NBNA score	ACME	−0.038	−0.18	0.14	0.58
	ADE	−0.220	−0.48	0.01	0.09
	Total Effect	−0.258	−0.54	0.05	0.11
PIH to NBNA score	ACME	−0.653	−0.98	−0.36	< 0.001 ***
	ADE	−0.305	−0.94	0.37	0.42
	Total Effect	−0.959	−1.79	−0.26	< 0.001 ***

***P < 0.001,**P < 0.01,*P < 0.05, Covariates: age, sex, GDM, and PIH

Abbreviations *GDM* Gestational Diabetes Mellitus, *PIH* Pregnancy-Induced Hypertension, BMI: Body Mass Index, *NBNA* Neonatal Behavioural Neurological Assessment, *GDS* Gesell Development Schedule, *ACME* Average casual mediation effect, *ADE* Average direct effect

Legend: Each panel presents the estimated ACME, ADE, and Total Effect for GDM and PIH on infant outcomes, using preterm birth as the mediator. Panels: a, GDM–GDS; b, GDM–BMI; c, GDM–NBNA; d, PIH–BMI; e, PIH–GDS; f, PIH–NBNA. Horizontal lines represent 95% confidence intervals; estimates to the left of the dashed zero line indicate negative associations. Abbreviations: GDM: gestational diabetes mellitus, PIH: pregnancy-induced hypertension, PTB: preterm birth, GDS: Gesell Development Schedule, NBNA: Neonatal Behavioural Neurological Assessment, BMI: body mass index.

## Discussion

Our study demonstrates that preterm birth plays a significant mediating role in the association between maternal diseases, specifically PIH and GDM, and infant growth and developmental outcomes, including NBNA score and BMI. Preterm birth exerted a complete negative mediating effect on BMI in the GDM cohort (ACME = − 0.0459) and on NBNA score in the PIH cohort (ACME = − 0.653). Within the NBNA assessment, subdomains were differentially affected among the 983 infants studied, with the greatest impairment observed in active muscle tone (34.28%), followed by behavioural ability (15.87%), passive muscle tone (3.36%), and primitive reflexes (0.10%).

These findings are consistent with previous studies reporting lower BMI in early infancy among GDM-exposed infants with a high risk of preterm birth [38, 39]. Previous research has also shown detrimental effects of PIH on the neurodevelopment of preterm infants. For example, infants of mothers with PIH exhibit reduced cerebral metabolism and oxygen consumption, which may lead to brain injury [40]. Preterm birth has also been identified with increased risk of brain injury through mechanisms such as brain hypoxia-ischemia, inflammation, and the need for mechanical ventilation [41–43]. However, prior studies typically examined GDM, PIH, and preterm birth as independent exposures, assessing their individual effects on infant growth or neurodevelopmental outcomes. The interaction between maternal diseases and preterm birth has been omitted, and the mediating effects of preterm birth were not explicitly identified. In our multivariate models, adjusting for preterm birth substantially weakened the associations of GDM and PIH with infant outcomes, reinforcing the conclusion that preterm birth is a key mediator. Exploring preterm birth as a mediator instead of an independent risk factor provides a novel perspective of interaction pathway linking maternal diseases and infant growth and neurodevelopment. It can provide insight into developing practical intervention to improve infant outcome.

Physiologically, PIH impairs placental perfusion, reducing the fatal supply of essential nutrients and oxygen to the foetus [44, 45]. This restriction contributes to the onset of preterm birth and can restrict intrauterine development. Consequently, preterm birth amplifies developmental challenges arising from shortened gestation and insufficient nutrient delivery, adversely affecting both neurological and physical growth [46]. Our results suggest that the active muscle tone, a subdomain of NBNA score, appears to be the most impaired neurodevelopmental function in preterm infants born to mothers with PIH. It has been revealed that PIH induced placental hypoperfusion can lead to hypoxia and oxidative stress [47], and experimental studies have also shown these processes facilitate placental inflammation and reactive oxygen species–mediated injury [48]. Such metabolic disturbances are likely to interfere with the maturation of neuromotor systems. In preterm birth infants, the corticospinal tracts, basal ganglia, and cerebellar circuits are not fully myelinated [49]. These neuromotor immaturity probably contributes to deficient active muscle tone.

Additionally, dysregulated glucose metabolism in GDM pregnancies has been previously revealed to affect infants’ growth via altered foetal insulin dynamics [50]. Maternal hyperglycaemia increases placental glucose transfer, stimulating compensatory proliferation and heightened secretory activity of foetal pancreatic β-cells and potentially resulting in sustained hyperinsulinaemia [50]. Given insulin’s anabolic properties, such exposure is considered to promote lipogenesis, protein synthesis, and somatic growth, thereby contributing to greater fat accretion and higher infants’ BMI at birth [51]. Following delivery, the abrupt cessation of maternal glucose supply, together with persistent β-cell hypersecretion, has been shown to predispose infants to hypoglycaemia [50]. Preterm infants are particularly vulnerable because of incomplete β-cell maturation, limited hepatic glycogen stores, reduced adipose depots, and immature gluconeogenic pathways, all of which constrain endogenous glucose production [52]. These mechanisms suggest that preterm birth may mediate the metabolic consequences of GDM by compromising infants’ glucose homeostasis and influencing BMI trajectories, thereby increasing the risk of later obesity and related disorders.

This study has several important strengths. First, the use of bootstrapping mediation analysis provides a novel method to investigate the relationship between preterm birth, maternal diseases, and infant growth and neurodevelopmental outcomes. This methodological approach is rarely applied in perinatal research and represents a clear statistical advancement. Second, blinded assessment of infant BMI, NBNA, and GDS by paediatric nurses minimised observer bias and ensured reliable outcome measurement. Third, the large cohort size and inclusion of multiple maternal conditions enhance the validity and generalisability of the findings and broaden understanding of prenatal risk factors. These strengths highlight the scientific value of the study and strengthen confidence in its conclusions.

However, this study has some limitations. This retrospective study was conducted at a single site which may limit the generalisability. Sociodemographic data such as education and income were incompletely collected because of limited participant disclosure, introducing potential interpretation bias. Classifications of preterm birth and maternal disease were relatively broad, and sample sizes for certain conditions, such as hyperthyroidism, were too small to support detailed mediation analysis. Other important maternal diseases cush as maternal chorioamnionitis and perinatal infection were also not included due to missing hospital records. A formal sensitivity analysis was not feasible considering the retrospective design, incomplete covariate data, and the small number in certain subgroups, which constrained alternative model testing. Future studies should address these issues through multi-centre prospective designs, larger and more precisely defined cohorts, and more comprehensive data-collection protocols to improve the precision and generalisability of findings.

The identification of preterm birth as a mediator between maternal disease and infant outcomes has several practical clinical implications. First, it highlights the need for early prenatal screening of mothers with conditions such as GDM and PIH, as timely monitoring and management may reduce the risk of preterm birth. Second, neonatal teams should consider maternal disease history when caring for preterm infants, who may be at heightened risk for impaired growth and neurodevelopment. Routine application of neurodevelopmental tools, including NBNA and GDS, may facilitate earlier identification of deficits and matching interventions. Finally, integrating maternal disease management with infants’ follow-up could enable more individualised medication care to improve outcomes for mother–infant pairs.

## Conclusions

This study indicates that preterm birth appears to be a mediator in the relationship between maternal diseases and infants’ growth and neurodevelopment. These findings support the integration of mediation analysis into future maternal-infant health research. It also emphasises the importance of early screening, targeted prenatal care, and timely neurodevelopmental assessments in clinical practice. Future research could extend this work with longitudinal and interventional designs to develop effective strategies that improve maternal and infant health outcomes.

## Supplementary Information

Supplementary material 1.

Supplementary material 2.

Supplementary material 3.

## Data Availability

The datasets used and/or analysed during the current study are available from the corresponding author on reasonable request.

## Abbreviations

PTB: Preterm Birth
GDM: Gestational Diabetes Mellitus
PIH: Pregnancy-Induced Hypertension
BMI: Body Mass Index
NBNA: Neonatal Behavioral Neurological Assessment
GDS: Gesell Development Schedule
