# Supplementary material for: Mediating role of preterm birth in the relationship between maternal disease and infant development
Source: BMC Pregnancy Childbirth. 2025 Nov 7;25:1174. doi: 10.1186/s12884-025-08268-7 (PMC12595764; doi:10.1186/s12884-025-08268-7)
Supplement: Supplementary file 1 — Supplementary material 1. [file 12884_2025_8268_MOESM1_ESM.docx]

**Children’s Health Survey Questionnaire**

**ID Number: _______**

Dear Parents,

We sincerely invite you to complete this child health questionnaire. Please respond based on the actual circumstances. By completing this questionnaire, you are providing informed consent to participate in this research on behalf of your child, and you agree to the use of both your and your child’s data for research purposes conducted by Xiangya Third Hospital.

| Please read the following statements and indicate whether you are willing/able to take part in the questionnaire:  − I have understood what is involved in taking part in this research, and how the information I provide will be used.  − Project findings may be published in various forms, including project reports, academic journal articles, conference papers and blog posts.  − I know who/where to ask any questions about the research or my participation.  − I am aware that I can withdraw from the study by closing my browser.  − I understand anything that can personally identify me will only be used for the purposes of analysing the data.  I have read all of the above statements and confirm that:  □ I am willing/able to voluntarily take part in the following questionnaire and support this research study.  □ I confirm that I am not willing/able to take part in the questionnaire |
| --- |

**Section I: Child’s Basic Information**

1. Child’s Age: _____ years _____ months

2. Sex: □ Male □ Female

3. Gestational age at birth: _____ weeks _____ days

4. Birth weight: _____ kg

6.Birth length/height: _____ cm

5. Delivery method: □ Vaginal delivery □ Caesarean section

6. Birth type: □ Singleton □ Twin/Multiple

7. Any special conditions before delivery:

□ Intrauterine hypoxia

□ Intrauterine growth retardation

□ Congenital malformation

□ Others: __________

8. Neonatal diseases (Multiple choice):

□ None

□ Premature birth

□ Neonatal pneumonia

□ Neonatal asphyxia

□ Meconium aspiration syndrome

□ Acute respiratory distress syndrome (Hyaline membrane disease)

□ Neonatal sepsis

□ Congenital heart disease

□ Hypoxic-ischemic encephalopathy

□ Intracranial infection

□ Intracranial haemorrhage

□ Neonatal haemolysis

□ Hypoglycaemia

□ Hypothyroidism

□ Pathological jaundice

□ Kernicterus

□ History of traumatic fracture

□ Others: _______________

9. Feeding method:

□ Breastfeeding

□ Formula feeding

□ Mixed feeding

**Section II: Mother’s Basic Information**

1.Mother’s age: ______ years

2. Mother’s height: ______ cm

3. Pre-pregnancy weight: ______ kg

Weight at delivery: ______ kg

4. Mother’s illnesses during pregnancy (Multiple choice):

□ None

□ Cold

□ Pneumonia

□ Anaemia

□ Thrombocytopenia

□ Diabetes or impaired glucose tolerance

□ Pregnancy-induced hypertension or eclampsia

□ Hypothyroidism

□ Hyperthyroidism

□ Allergic diseases

□ Intrahepatic cholestasis of pregnancy

□ Heart disease

□ Rubella

□ Syphilis

□ Infectious diseases

□ Genetic diseases

□ Schizophrenia/Depression and other mental disorders

□ Others: _____________
