# Supplementary material for: Mediating role of preterm birth in the relationship between maternal disease and infant development
Source: BMC Pregnancy Childbirth. 2025 Nov 7;25:1174. doi: 10.1186/s12884-025-08268-7 (PMC12595764; doi:10.1186/s12884-025-08268-7)
Supplement: Supplementary file 2 — Supplementary material 2. [file 12884_2025_8268_MOESM2_ESM.docx]

**Neonatal Behavioral Neurological Assessment (NBNA)**

**20 Items:** Total Score: ______ points

| **Category** | **Test Condition** | **Score 0** | **Score 1** | **Score 2** | **Age (days)** | | | |
| --- | --- | --- | --- | --- | --- | --- | --- | --- |
|  |  |  |  |  | 2~3 | 5~7 | 12~14 | 26~28 |
| Behavioral Ability |  |  |  |  |  |  |  |  |
| 1. Light Habituation | Sleep | ≥11 | 7–10 | ≤6 |  |  |  |  |
| 2. Sound Habituation | Sleep | ≥11 | 7–10 | ≤6 |  |  |  |  |
| 3. Response to Rattle Sound | Quiet Awake | No head/eye movement | Head or eye turn <60° | Head or eye turn ≥60° |  |  |  |  |
| 4. Response to Speaking Face | Quiet Awake | No head/eye movement | Head or eye turn <61° | Head or eye turn ≥61° |  |  |  |  |
| 5. Response to Red Ball | Quiet Awake | No head/eye movement | Head or eye turn <62° | Head or eye turn ≥62° |  |  |  |  |
| 6. Consolability | Crying | Cannot console | Difficult | Easily or spontaneously consoled |  |  |  |  |
| Passive Muscle Tone |  |  |  |  |  |  |  |  |
| 7. Scarf Sign | Awake | Wraps around neck | Elbow crosses midline | Elbow doesn't reach midline |  |  |  |  |
| 8. Arm Recoil | Awake | None | Weak/slow, >3s | Active, repeatable, ≤3s |  |  |  |  |
| 9. Popliteal Angle | Awake | >110° | 90–110° | <90° |  |  |  |  |
| 10. Leg Recoil | Awake | None | Weak/slow, >3s | Active, repeatable, ≤3s |  |  |  |  |
| Active Muscle Tone |  |  |  |  |  |  |  |  |
| 11. Head Control* | Awake | Absent/abnormal | Difficult | Good, head held upright for ≥1–2s | ~ |  |  |  |
| 12. Grasp | Awake | None | Weak | Good, repeatable |  |  |  |  |
| 13. Traction Response | Awake | None | Lifts part of the body | Lifts the whole body |  |  |  |  |
| 14. Supporting Reaction (standing) | Awake | None | Incomplete, brief | Strong, supports full body |  |  |  |  |
| Primitive Reflexes |  |  |  |  |  |  |  |  |
| 15. Stepping/Placing Reflex | Awake | None | Difficult to elicit | Good, repeatable |  |  |  |  |
| 16. Moro Reflex | Awake | None | Weak/incomplete | Good, repeatable |  |  |  |  |
| 17. Sucking Reflex | Awake | None | Weak | Good, synchronized with swallowing |  |  |  |  |
| General Assessment |  |  |  |  |  |  |  |  |
| 18. Alertness | Awake | Coma | Drowsy | Normal |  |  |  |  |
| 19. Crying | Crying | Absent | Weak, sharp, or excessive | Normal |  |  |  |  |
| 20. Activity Level | Awake | Absent or excessive | Reduced or increased | Normal |  |  |  |  |

**Scoring criteria**

The Neonatal Behavioral Neurological Assessment (NBNA) is a comprehensive 20-item scale designed to evaluate the neurological and behavioral integrity of full-term neonates. The method was originally developed by Professor Bao in 1990, integrating the Brazelton Neonatal Behavioral Assessment Scale (NBAS) and the Amiel-Tison neuromotor assessment, alongside clinical insights specific to Chinese newborn populations.

**1. Items**
Each item is rated on a 0-2 point scale, with higher scores indicating more favorable neurological function.
- Total possible score: 40 points
- Interpretation:
 - Normal: 35–40 points
 - Abnormal: <35 points

**2. Eligibility and Examination Conditions**

a. Applicable population: Full-term neonates (≥37 gestational weeks); preterm infants should be assessed at corrected gestational age ≥40 weeks.
b. Timing: Between two feedings, when the neonate is naturally awake or can be aroused.
c. Environment:
 - Quiet, semi-dark room
 - Room temperature: 22°C to 27°C
 - No external disturbances
d. Personnel: Certified senior neonatal nurses who have completed standardized NBNA training.
e. Duration: Examination must be completed within 10 minutes to ensure consistency.
