# Supplementary material for: Mediating role of preterm birth in the relationship between maternal disease and infant development
Source: BMC Pregnancy Childbirth. 2025 Nov 7;25:1174. doi: 10.1186/s12884-025-08268-7 (PMC12595764; doi:10.1186/s12884-025-08268-7)
Supplement: Supplementary file 3 — Supplementary material 3. [file 12884_2025_8268_MOESM3_ESM.docx]

**Gesell Developmental Assessment Table**

| Age | Gross Motor Skills | Fine Motor Skills | Adaptive Skills | Language | Personal-Social |
| --- | --- | --- | --- | --- | --- |
| 4 weeks (0.93 months) | Supine: Head turns to one side predominantly Pull-to-sit: Full head lag Prone: Slight head lift Prone: High hips, leg wriggling | Suspended ring: Tightened grasp when touching a rattle Supine: Hands clenched | Tracks rattle within vision Brief retention of rattle Decreased activity when hearing a bell | Expression: Blank or short gaze Vocalization: Soft throat sounds | Gaze around vaguely Reduced activity when observing the examiner Night feeding twice |
| 8 weeks (1.87 months) | Supine: Head at midline Symmetrical posture Prone: Repeated head lift to 45° | Maintains grasp on a suspended ring | Slow attention to midline Tracks beyond midline Vertical tracking begins | Expression: More alert appearance Cooing sounds | Repeated gaze at examiner's face Responsive smile Night feeding once |
| 12 weeks (2.8 months) | Supine: Head mostly in center Sitting: Slight forward tilt of head, slightly unstable Prone: Prolonged head lift, automatic forearm support | Hands open or lightly clenched Begins grabbing clothes or objects | Immediate attention at midline Grasps rattle and looks at it Smooth vertical tracking | Expression: Deep breathing when excited Laughing out loud | Anticipates feeding by looking at caregiver Looks at hands, brings hands together |
| 16 weeks (3.7 months) | Supine: Hands clasped Sitting: Head stable when leaning forward Prone: Raises head to 90°, rolls from prone to supine | Actively grasps rattle, touches table surface | Follows moving objects 180° Puts rattle in mouth Reaches with empty hand | Begins to laugh audibly Screeches | Touches bottle during feeding Smiles spontaneously at people |
| 20 weeks (4.7 months) | Rolls from supine to prone Sits with head upright and stable Supports body weight with legs | Grasps wooden blocks with fingers | Grasps nearby rattle Searches for dropped toys | Vocalizes to people and toys Turns head toward sound | Puts objects in mouth Shows preference for familiar people |
| 24 weeks (5.6 months) | Sits with minimal support Attempts crawling motions | Grasps objects with entire hand Removes cloth from face | Holds two objects simultaneously Reaches for third object Notices falling toys | Babbles two syllables Laughs during play | Feeds on solid foods Plays alone with toys |
| 28 weeks (6.5 months) | Sits upright for 1 minute Pulls feet to mouth when supine Begins rotating around the abdomen | Grasps small balls with palmar grasp Attempts to rake objects | Grasps and shakes rattle actively Transfers objects between hands Pays sustained attention to falling toys | Babbling with multiple syllables e.g., "da-da", "ba-ba" | Reaches for distant toys Plays by mouthing toys |
| 32 weeks (7.5 months) | Sits steadily for over 10 minutes Stands holding onto rail without help | Radial palmar grasp develops | Attempts to retrieve two blocks Pulls a string to get a ring | Imitates coughing and tongue sounds Responds to familiar people's voices | Distinguishes strangers from familiar people Shows excitement during social play |
| 36 weeks (8.4 months) | Transitions from sitting to prone with control Crawls using hands and knees | Begins scissor grasp Rakes small balls | Places two blocks together Lifts cup to mouth Searches for hidden objects | Says first meaningful words Laughs during music | Feeds self with biscuits Engages in peek-a-boo |
| 40 weeks (9.3 months) | Stands alone briefly Moves along rail while holding it | Pincer grasp develops Picks up small items quickly | Places toys in a cup Pulls strings intentionally | Understands simple phrases Names familiar objects | Feeds self with cup Imitates simple actions |
| 44 weeks (10.3 months) | Walks holding one hand Squats to pick up objects | Places blocks down clumsily Grasps rope quickly | Places blocks inside a cup Recovers hidden toys | Combines two words Understands simple commands | Helps dress by extending limbs Waves goodbye |
| 48 weeks (11.2 months) | Stands alone steadily Takes steps holding one hand | Precise pincer grasp for small balls Throws ball lightly | Stacks two blocks Searches for missing toys | Names objects during picture reading Imitates animal sounds | Feeds self with spoon Offers toys to others |
| 52 weeks (12 months) | Walks a few steps alone Climbs stairs with assistance | Attempts to build towers | Puts blocks into a container Searches for hidden toys | Uses ~8 words meaningfully Points to body parts when asked | Shows affection to family Enjoys playing with others |

**Gesell Developmental Scale**

Overall Developmental Quotient (DQ): ______

Adaptive Behavior: ______

Gross Motor Skills: ______

Fine Motor Skills: ______

Language: ______

Personal-Social Skills: ______

**Scoring criteria**

The Gesell Developmental Schedule (GDS) is a standardized psychological assessment tool used to evaluate the developmental levels of children aged 0 to 6 years. It is also one of the key diagnostic methods for identifying intellectual disabilities in this age group. The scale was introduced to China and standardized domestically in the 1970s by Professor Lin.
The GDS evaluates a child's neurological maturity and functional development based on behavioral patterns observed in typically developing children. Developmental performance is quantified by:
- Developmental Age: The age-equivalent level at which the child demonstrates typical developmental abilities.
- Developmental Quotient: A numerical expression of the child's developmental level relative to their chronological age.

**Scoring Method**

1. Developmental Quotient Calculation

DQ = (Developmental Age / Chronological Ag) x 100
- Developmental Age: Derived from the highest developmental milestones the child achieves within each domain, using specific scoring formulas for different age intervals. The scale provides four categories of calculation formulas, adapted according to the child's age group.
- Chronological Age: Actual age in months.

1. Domain Scores & Total Score

Each domain is assessed independently. The total DQ score can be interpreted as a comprehensive indicator of the child’s global developmental level. Domain-specific DQ values can help pinpoint areas of developmental delay.

**Interpretation of Developmental Quotients**

| Developmental Quotient | Developmental Level |
| --- | --- |
| ≥ 85 | Normal development |
| 75 – 84 | Borderline |
| 55 – 74 | Mild developmental delay |
| 40 – 54 | Moderate developmental delay |
| 25 – 39 | Severe developmental delay |
| < 25 | Profound developmental delay |
